# Detecting Conditional Dependence Using Flexible Bayesian Latent Class Analysis

**DOI:** 10.3389/fpsyg.2020.01987

**Published:** 2020-08-06

**Authors:** Jaehoon Lee, Kwanghee Jung, Jungkyu Park

**Affiliations:** ^1^Department of Educational Psychology and Leadershi, Texas Tech University, Lubbock, TX, United States; ^2^Department of Psychology, Kyungpook National University, Daegu, South Korea

**Keywords:** conditional dependence, Bayesian latent class analysis, approximate independence, prior variance, model fit

## Abstract

A fundamental assumption underlying latent class analysis (LCA) is that class indicators are conditionally independent of each other, given latent class membership. Bayesian LCA enables researchers to detect and accommodate violations of this assumption by estimating any number of correlations among indicators with proper prior distributions. However, little is known about how the choice of prior may affect the performance of Bayesian LCA. This article presents a Monte Carlo simulation study that investigates (1) the utility of priors in a range of prior variances (i.e., strongly non-informative to strongly informative priors) in terms of Type I error and power for detecting conditional dependence and (2) the influence of imposing approximate independence on model fit of Bayesian LCA. Simulation results favored the use of a weakly informative prior with large variance–model fit (posterior predictive p–value) was always satisfactory when the class indicators were either independent or dependent. Based on the current findings and the additional literature, this article offers methodological guidelines and suggestions for applied researchers.

## Introduction

Latent class analysis (LCA; Lazarsfeld and Henry, 1968) is a probability model–based tool that analyzes categorically scored data by introducing a latent variable. As the name suggests, the latent variable (usually) consists of a small number of levels, called “latent classes” that characterize the categories of a theoretical construct. The primary aim of LCA is to identify class members that are homogenous within the same class but distinct between different classes in terms of responses to a set of observed variables (i.e., latent class indicators). Once identified, the latent classes are compared with each other for auxiliary variables such as covariates and distal outcomes presumed to be antecedents or consequences of the classification (Asparouhov and Muthén, 2014; Vermunt, 2010).

LCA has been extended to accommodate various types of observed data–for example, latent profile analysis with continuous indicators, multilevel mixture models for clustered data, growth mixture models and latent transition models for longitudinal observations, and survival mixture models with time–censored indicators. Owing to such flexibility in data distribution that can be modeled, LCA and other mixture approaches recently have been increasingly adopted in a variety of disciplines, including cognitive diagnostic testing (Rupp et al., 2010) health and medicine (Schlattmann, 2010) genetics (McLachlan et al., 2004) machine learning (Yang and Ahuja, 2001) and economics (Geweke and Amisano, 2011). More recently, methodological advances have made it feasible to estimate LCA models within the Bayesian framework; see Li et al. (2018) and Asparouhov and Muthén (2010).

### Assumption of Conditional Independence in LCA

Another key advantage of using LCA is that it does not require the rigid assumptions of traditional classification methods (Muthén, 2002; Muthén and Shedden, 1999; Magidson and Vermunt, 2004). Still, LCA assumes that class indicators are conditionally independent of each other, given class membership–i.e., class indicators are uncorrelated within each class. This implies that the associations among the indicators are accounted for only by the latent classes and there are no other latent variables influencing the indicators. A violation of this local independence assumption (i.e., *conditional dependence*) can lead to severe bias in estimating LCA parameters that include classification error, class probabilities, and posterior classification probabilities (Vacek, 1985; Torrance-Rynard and Walter, 1998; Albert and Dodd, 2004). An additional drawback that is common with conditional dependence is model misfit. That is, unmodeled dependence among indicators can induce poor model fit and incorrect values of information criteria [e.g., Akaike information criterion (AIC), Bayesian information criterion (BIC)], resulting in spurious latent classes (usually with an overestimated number of classes).

### Current Methods for Handling Conditional Dependence

When conditional dependence is suspected, a viable option is to model the dependence “directly” (Uebersax, 1999; Hagenaars, 1988). The correlation between each pair of indicators is freely estimated; and a significant improvement in model fit supports relaxing the conditional independence assumption on locally dependent indicator pairs. Still, there is a caveat to this approach. Freeing the constrained parameters (correlations) often makes the model non–identifiable or results in highly unstable parameter estimates, because the dependencies captured by the latent classes are difficult to separate from nuisance local dependencies. An alternative option to deal with conditional dependence is employing a latent variable(s). *Factor* mixture modeling, for example, models conditional dependence by allowing for the indicators to be loaded on a continuous latent variable in addition to their loading on the discrete latent variable representing/forming classes (Lubke and Muthén, 2007). This approach is yet limited because in many applications indicators do not necessarily represent an interrelated dimension(s) of a generic construct and thus models can suffer from estimation challenges. Other applications of modeling conditional dependence can be found in Qu et al. (1996), Wang and Wilson (2005), Im (2017), Hansen et al. (2016), and Zhan et al. (2018).

Beyond handling conditional dependence, researchers may want to monitor and detect the sources of conditional dependence. Magidson and Vermunt (2004) proposed bivariate residual (BVR)–a high value of BVR for a pair of indicators reveals model misfit due to (residual) conditional dependence between the indicators. A drawback of BVR is that its distribution is unknown. Oberski et al. (2013) recommended using BVR with a bootstrapping procedure which approximates a chi–square distribution. They also showed that Lagrange multiplier test, also called modification index, performs well in identifying the sources of conditional dependence, showing adequate power and controlled Type I error.

### New Method: Assumption of Conditional Independence in Bayesian LCA

In Bayesian statistics, the researcher’s belief about the value of a parameter is formulated into a distribution, which is called *prior distribution* (often simply called *prior*). Data also inform about the parameter value, yielding a conditional distribution of the data given the parameter, which is called *likelihood*. The likelihood modifies the prior distribution into a *posterior distribution* (often simply called *posterior*). Finally, a parameter estimate is inferred through a sampling of ‘plausible’ values from the posterior.

A prior having small variance (i.e., a narrow prior distribution) represents the researcher’s small uncertainty about the parameter value. This small–variance (“informative”) prior makes relatively more contribution to constructing the posterior than does the likelihood. On the other hand, a prior having a large variance (i.e., a wide prior distribution) represents large uncertainty about the parameter value, and the large–variance (“non–informative”) prior yields relatively less influence on the formation of the posterior than does the likelihood (Muthén and Asparouhov, 2012; MacCallum et al., 2012). Thus, the model would fit the data very closely if non–informative priors were specified for all model parameters, but the parameter estimates might be scientifically untenable (Gelman, 2002).

Recent methodological advances have made it possible to incorporate latent variable modeling in the framework of Bayesian statistics (O’sullivan, 2013; Silva and Ghahramani, 2009). Bayesian estimation in latent variable modeling is advocated particularly for avoiding the likely problem of a non–identifiable model or an improper solution in maximum–likelihood estimation. For instance, the researcher may replace the parameter specification of “exact zeros” with “approximate zeros” by imposing informative priors on the parameters that would have been fixed to 0 for hypothesis testing or scale setting in ML estimation (Muthén and Asparouhov, 2012). In Muthén and Asparouhov (2012) illustration of Bayesian structural equation modeling (BSEM), priors for factor loadings are specified to be normal with zero mean and infinity variance (i.e., non–informative priors), while priors for cross–loadings are specified to follow a normal distribution having zero mean and 0.01 variance (i.e., informative priors)–95% of the cross–loading values are between –0.2 and 0.2 in the prior distribution. A few real–data applications and Monte Carlo simulations showed that the “approximate zeros” strategy performs well for both measurement and structural models that involve cross–loadings, residual correlations, or latent regressions with respect to model fit testing, coverage for key parameters, and power to detect model misspecifications (Muthén and Asparouhov, 2012).

The idea of this “approximate zeros” approach is applicable for detecting and accommodating violations of the conditional independence assumption in LCA. Rather than fixing to 0, the researcher may freely estimate all or some tetrachoric (for binary class indicators) or polychoric (for polytomous class indicators) correlations among the indicators using informative priors with zero mean and small variance (i.e., *approximate independence*; Asparouhov and Muthén, 2011). Asparouhov and Muthén (2010, 2011) suggested Bayesian LCA that relaxes the conditional independence assumption to an assumption of approximate independence. This flexible Bayesian LCA can avoid a false class formation that is often caused by ignoring conditional dependence, or equivalently, neglecting (i.e., fixing) nonzero correlations among indicators.

To illustrate this method, a model was fitted to the data from Midlife in the United States (MIDUS), 2004—-2006, a national survey of 4,963 Americans aged 35 to 86. The class indicators were 10 binary items (*yes*/*no*) asking the main reasons for discrimination respondents experienced: age, gender, race, ethnicity or nationality, religion, height or weight, some other aspect of appearance, physical disability, sexual orientation, and some other reason (B1SP3A–B1SP3J). The model specified two classes and approximate independence between each pair of the indicators by imposing a prior on the tetrachoric correlation. The model fit was adequate, and the classification quality was excellent with an entropy value of 1 (35% of the sample in the first class and 65% in the second class). More important, the correlations among the indicators deviated from zero ranging from –0.28 to 0.42 and they were higher in the first class (*M* = 0.19, SD = 0.14) than in the second class (*M* = –0.05, SD = 0.15). These results suggest that the model of approximate independence could detect and properly model the fair amounts of conditional dependence. The M*plus* input for this analysis is shown in Appendix (B1SP3A-B1SP3J are renamed to U1-U10 for illustrative purpose).

More research is warranted to investigate the performance of this method as a tool for detecting conditional dependence and acknowledge potential consequences of incorporating approximate independence into LCA models. Another scientific inquiry is evaluating model fit of Bayesian LCA under the assumption of approximate independence. Asparouhov and Muthén (2011) argued that posterior predictive checking is needed to evaluate the fit of flexible Bayesian LCA models. In Bayesian statistics, one possible measure of model-data fit is *posterior predictive p-value* (PPP). The process of deriving PPP is quite technical (Gelman et al., 1996) but the key application is simple-a PPP around 0.50 suggests an excellent fit. Although there is no theoretical cutoff of alarming poor fit, Muthén and Asparouhov (2012) suggested that a PPP around 0.10, 0.05, or 0.01 would indicate a significantly ill-fitting model.

### Purpose of Study

Although the literature has suggested flexible Bayesian LCA as an alternative approach to detect and accommodate conditional dependence between indicators, there is no specific guideline about to what degree a prior should be informative to properly model the conditional dependence (Ulbricht et al., 2018). To the authors’ knowledge, only a strongly informative prior was empirically studied under limited conditions (Asparouhov and Muthén, 2011). Thus, this article presents a simulation study of which aim is to examine (1) the utility of priors in flexible Bayesian LCA with a wide range of variances (i.e., strongly non-informative to strongly informative priors) in terms of Type I error and power for detecting conditional dependence; and (2) the influence of imposing approximate independence on fit (PPP) of Bayesian LCA. The current investigation focuses on the simple case of binary indicators measured in cross-sectional research-that is, flexible Bayesian LCA as a beginning attempt to understand the performance of LCA under the assumption of approximate independence.

## Materials and Methods

This section illustrates the model specifications of LCA; and describes the simulated conditions and Monte Carlo procedure utilized to examine the performance of Bayesian LCA under the approximate independence assumption.

### Bayesian LCA Models

Let *Y* be a full response vector for a set of *J* indicators, where *j* = 1,…, *J*; and let *X* be a discrete latent variable consisting of *M* latent classes. A particular class is denoted by *m*. The probability of a particular response pattern on *J* indicators can be defined as follows:

(1) $$P\left(Y\right)=\sum_{m=1}^{M}P\left(X=m\right)f\left(Y|X=m\right)$$

Let *y*_*j*_ denote a response on indicator *j*; then conditional density for indicator *j* (*f*(*y*_*j*_|*X* = *m*)) is statistically independent of each other, given latent class membership *m*. Therefore, the conditional independence assumption can be represented as

(2) $$P\left(Y\right)=\sum_{m=1}^{M}P\left(X=m\right)\prod_{j=1}^{J}f\left(y_{j}|X=m\right)$$

The conditional density *f*(*y*_*j*_|*X* = *m*)depends on the assumed distribution of responses. Suppose the response vector *Y* = (*y*_1_,*y*_2_,…,*y*_*J*_)^*T*^consists of *J* binary variables. Then the *m*th latent class density is given by $f\left(y_{j}|X=m\right)=\rho_{m\,j}^{y_{j}}{\left(1-\rho_{m\,j}\right)}^{1-y_{j}},$ where ρ_*mj*_ denotes the probability of endorsing item *j* for given latent class membership *m* (*f*(*y*_*j*_ = 1|*X* = *m*)).

This standard LCA model can be formulated in terms of a multivariate probit model with a continuous latent response variable $y_{j}^{*}$ for indicator *j*:

(3) $$y_{j}^{*}|X∼N\,\left(\mu_{j\,m},1\right),$$

where*y*_*j*_ = 0, then$y_{j}^{*}<0$; thus ρ_*mj*_ = 0, then $P\left(y_{j}^{*}<0|X=m\right)=\Phi \left(\mu_{j\,m}\right)$. A multivariate form of this model can be expressed as

(4) $$y_{j}^{*}|X∼N\,\left(\mu_{j\,m},1\right),$$

where $Y^{*}={\left(y_{1}^{*},y_{2}^{*},\ldots ,y_{J}^{*}\right)}^{T}$, μ_*m*_ = (μ_1*m*_,μ_2*m*_,…,μ_*Jm*_)^*T*^ and *I* is a correlation matrix that all the off-diagonal elements are equal to 0. The conditional dependence model with correlated indicators is to replace Eq. 4 with

(5) $$Y^{*}|X∼N\,\left(\mu_{m},\Sigma_{m}\right),$$

where Σ_m_ represents an unrestricted correlation matrix. The off-diagonal elements in Σ_m_ are called a tetrachoric correlation for binary indicators that can be varied across classes. When indicators have more than two categories, the correlation is called a polychoric correlation. These correlations can be estimated by maximizing the log-likelihood of the multivariate normal distribution. The parameters of the conditional dependence LCA model can be estimated using the Markov chain Monte Carlo (MCMC) algorithm. The details about MCMC estimation with prior information on each parameter are provided by Asparouhov and Muthén (2011).

### Population Model (Data Generation)

Two classes (Class 1 and Class 2) of equal size were simulated in the population model with 10 binary class indicators. The simulation conditions examined by Asparouhov and Muthén (2011) were included in the current study for the sake of comparability, along with some additional conditions. To vary the level of conditional dependence among the indicators, data were generated such that the tetrachoric correlation matrix in Class 1 had all zero values (no dependence); or three nonzero values ρ_1,12_ = ρ_1,39_ = ρ_1,57_ = 0.20, 0.50, or 0.80 (small, medium, and large, respectively) and zero values for all other elements. Here, ρ_*m,jk*_ is the correlation between indicator *j* and indicator *k* in class *m*. The tetrachoric correlation matrix in Class 2 had all zero values; or all zero values except for ρ_*2,46*_ = 0.20, 0.50, or 0.80. The size of the nonzero correlations, if present, were matched to be equal between the two classes. In addition, the indicator thresholds μ_*m,k*_ were set to be equal within each class but opposite in sign between the two classes-μ_*1,k*_ = 1.00 and μ_*2,k*_ = −1.00, which yields a reasonable class separation at two standard deviations (Lubke and Muthén, 2007). Sample size was also simulated as *N* = 50, 75, 100, 500 in increments of 25, and *N* = 1,000.

### Analysis Model

The analysis model specified a weakly informative prior for the indicator thresholds μ_*m,k*_ (∼ *N* [0, 5]) and for the class threshold *q*_*m*_ (∼ Dirichlet distribution *D* [10, 10]). They are the default priors in M*plus* 8 and not the focus of the current study. Another default prior, inverse Wishart distribution *IW* (*I*, *f*), where *I* is identity matrix and *f* is degrees of freedom (*df*), was specified for the tetrachoric correlations among the class indicators. To vary the variance of the priors, *f* was set to be 11, 52, 108, 408, or 4,000. In this way, the correlations were modeled as following a symmetric beta distribution on the interval [–1, 1] with mean zero and variance of 0.33, 0.02, 0.01, 0.003, or 0.00003-consequently, 95% confidence limits of the correlations approximately equal to ±1.13, ±0.30, ±0.20, ±0.10, or ±0.01, respectively (Barnard et al., 2000; Gill, 2008; Asparouhov and Muthén, 2011). The prior having the largest variance (0.33) was strongly non-informative because it indeed corresponds to a uniform distribution on the interval [–1, 1]. The prior having the second largest variance (0.02) was considered weakly non-informative, and the other three priors with relatively small variance were considered strongly informative (0.00003), informative (0.003), and weakly informative (0.01).

### Monte Carlo Specifications

Two hundred samples were drawn from each of 400 simulation conditions (20 sample sizes × 4 levels of correlations among the indicators × 5 prior variances), yielding a total of 80,000 replications. In Bayesian estimation, two independent Markov chains created approximations to the posterior distributions, with a maximum of 50,000 iterations for each chain. The first half of each chain was discarded as being part of the burn-in phase. Convergence was assessed for each parameter using the Gelman-Rubin criterion (Gelman and Rubin, 1992) the convergence rate was 100% in all simulated conditions. The medians of the posterior distributions were reported as Bayesian point estimates, which is the default setting in M*plus* 8. Model fit (PPP) was calculated based on the chi-square discrepancy function (Scheines et al., 1999; Asparouhov and Muthén, 2010).

## Results

This section presents the results of the simulation study regarding (1) the effects of the condition factors (sample size, correlation size, prior variance) on Type I error and power of flexible Bayesian LCA for detecting conditional dependence; (2) the effects of the condition factors on fit (PPP) of the model; and (3) bias in Bayesian estimates of indicator correlations.

### Type I Error and Power for Testing Conditional Dependence

**FIGURE 1:**
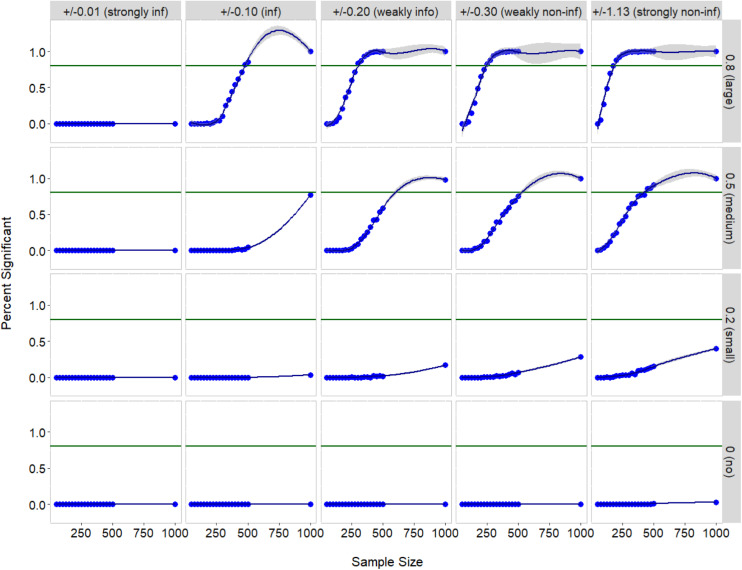
Accuracy of testing zero and nonzero correlations by correlation size, sample size, and prior variance. The blue dots represent average % significant and the blue line represents smoothed conditional means of % significant. The green line indicates % significant of 0.80, a satisfactory power to detect a true nonzero correlation (top three panels). Note that the conditional means greater than 1 are not plausible values and merely indicate prediction artifact.

Figure 1 depicts how often (zero or nonzero) correlations were detected to be significantly different from zero at the nominal alpha level of 0.05-“% significant.” It should be noted that this figure summarizes the outcomes on a particular pair of indicators (the first and second indicators at Class 1), but the results are almost identical to those from other indicator pairs. Type I error, false positive on a true zero correlation, was well controlled in the flexible Bayesian LCA model. In fact, the average % significant, represented by the blue lines in Figure 1, was below 5% for any prior variance and for any sample size (see the bottom panel).

Power for detecting a nonzero correlation increased with the use of less informative priors (see the top three panels). As would be anticipated, a true nonzero correlation (ρ_*1,12*_ = 0.20, 0.50, or 0.80) was seldom estimated to be significantly different from zero if the strongly informative prior was imposed on this parameter. Also, power for detecting a true small correlation (ρ_*1,12*_ = 0.20) was always less than satisfactory (i.e., <80%) regardless of prior variance (see the bottom second panel). When the correlation was moderate (ρ_*1,12*_ = 0.50), a (either weakly or strongly) non-informative prior and a sample size of at least 500 were required to yield acceptable power. For the prior that allows for 95% of estimates to be within +0.10 and –0.10 (i.e., an informative prior), power was adequate (≥80%) when and only when a true large correlation (ρ_*1,12*_ = 0.80) was estimated from a sample greater than *N* = 500. For the priors having larger variance (weakly informative, non-informative, and strongly non-informative priors), power for detecting a true large correlation (ρ_*1,12*_ = 0.80) was satisfactory if the sample size was at least 300.

### Model Fit of Flexible Bayesian LCA

Analysis of variance was conducted to identify which condition factors considerably influenced PPP. The estimated effect sizes (η^2^) of the three condition factors and their interactions are provided in Table 1. Sample size had a negligible effect on PPP (η^2^ = 0.020), which is similar to the findings for Bayesian confirmatory factor analysis in Muthén and Asparouhov (2012). PPP was largely influenced by the size of correlations (i.e., the magnitude of conditional dependence) among the indicators (η^2^ = 0.388), as well as by the choice of prior variance for these parameters (η^2^ = 0.104). Small to moderate effects were observed for the interactions between the condition factors (η^2^ = 0.014–0.057).

**TABLE 1:** Effects of simulation condition factors on posterior predictive *P*-value.

Condition factor	η^2^
Sample size (N)	0.020
Correlation size (C)	0.388
Prior variance (P)	0.104
N × C	0.042
N × P	0.022
C × P	0.057
N × C × P	0.014

**FIGURE 2:**
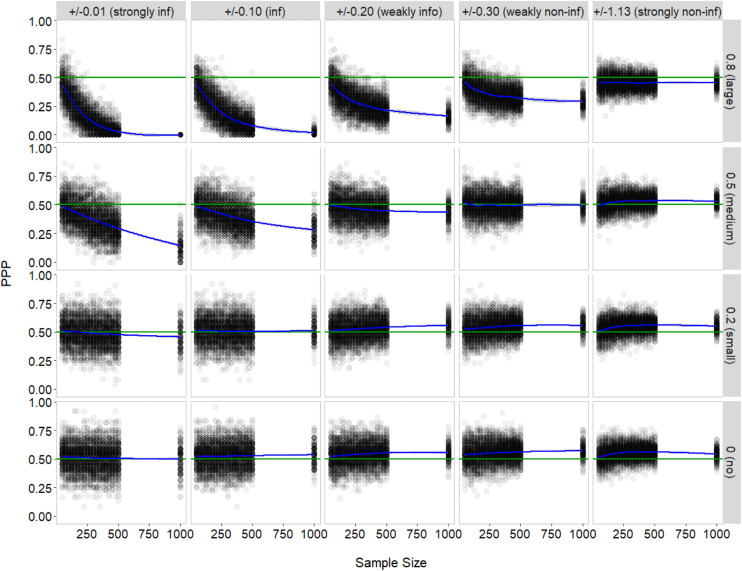
Posterior predictive *P*-value by sample size, correlation size, and prior variance. The gray dots represent estimated PPP and the blue line represents smoothed conditional means of PPP. The green line indicates PPP of 0.50, an excellent fit.

Figure 2 describes the (large) effects of correlation size and prior variance in a series of plots, in which the *y*-axis represents PPP and the *x*-axis represents sample size. Recall that the tetrachoric correlations between the indicators were simulated to be 0, 0.20, 0.50, or 0.80-no, small, medium, and large, respectively (in Figure 2, from the bottom to top panels). Also, recall that the prior (symmetric beta) distributions for these parameters were specified to have mean zero and variance of 0.00003, 0.003, 0.01, 0.02, or 0.33-strongly informative, informative, weakly informative, weakly non-informative, and strongly non-informative, respectively (in Figure 2, the panels from left to right). The average PPP, represented by the blue lines in Figure 2, was close to its expected value of 0.50 when the actual value of the correlations was zero-that is, when the indicators were conditionally independent within each class-regardless of different choices for prior variance. Deviations from 0.50 appeared with the use of less informative priors (i.e., larger prior variances), though the discrepancy was negligible (see the bottom panel). Similar results were found in the case of small correlations, or equivalently, small conditional dependence (ρ_*m,jk*_ = 0.20; see the second bottom panel). In contrast, the average PPP decreased farther from 0.50 with the correlations greater than small (ρ_*m,jk*_ = 0.50–0.80; see the top two panels), and more quickly when a more informative prior was chosen for the correlations-that is, interaction between correlation size and prior variance. Still, model fit was good if the strongly non-informative prior was specified for moderate and large correlations (in fact, correlations in any size).

The interactions of sample size with correlation size and prior variance are also exhibited in Figure 2. When the correlations between the indicators were less than moderate (ρ_*m,jk*_ = 0–0.20; see the bottom two panels), sample size had no impact on model fit. When the correlations were rather moderate or large (ρ_*m,jk*_ = 0.50–0.80; see the top two panels), PPP decreased as sample size increased; such deterioration in model fit became greater as the prior was more informative. In general, PPP was less variable as compared to the findings for Bayesian CFA (Muthén and Asparouhov, 2012).

### Bias in Bayesian Estimates Due to the Presence of Conditional Dependence

Table 2 shows the Bayesian estimates of indicator correlations in Class 1 from the fitted flexible Bayesian LCA models. Recall that the correlation between the first and second indicators was simulated to beρ_*1,12*_ = 0, 0.20, 0.50, or 0.80, while the correlations between the first and third indicators and between the third and eighth indicators were always zero in the population (ρ_1,13_ = ρ_1,38_ = 0). These three indicators were purposefully selected to scrutinize the influence of conditional dependence on estimating correlations among other pairs of indicators. Similar to the findings for cross-loadings in Bayesian CFA (Asparouhov and Muthén, 2011) the estimate of a true zero correlation (i.e., conditional independence) was negatively biased by the presence of a true nonzero correlation(s) (i.e., conditional dependence). For example, both ${\hat{\rho }}_{1,13}$ and ${\hat{\rho }}_{1,38}$ deviated farther from their population value (0) in the negative direction as ρ_*1,12*_ increased. Such bias became greater (i) when a less informative prior was specified for these parameters and (ii) when one (or both) of the two indicators had a nonzero correlation with other indicator(s) within the same class. Still, Type I error was well controlled-a true zero correlation (ρ_1,13_ = ρ_1,38_ = 0) was estimated as significantly different from zero in less than 5% of chances.

**TABLE 2:** Bayesian estimates of true zero correlations between class indicators.

ρ_*1,12*_	Prior	${\hat{\rho }}_{1,12}$	${\hat{\rho }}_{1,13}$	${\hat{\rho }}_{1,38}$
0.20	Strongly informative	0.0002	0.0000	0.0000
	Informative	0.0134	–0.0006	0.0008
	Weakly informative	0.0385	–0.0022	0.0019
	Weakly non-informative	0.0594	–0.0036	0.0027
	Strongly non-informative	0.1012	–0.0077	0.0052
0.50	Strongly informative	0.0004	0.0000	0.0000
	Informative	0.0370	–0.0006	0.0000
	Weakly informative	0.1023	–0.0024	–0.0002
	Weakly non-informative	0.1544	–0.0040	–0.0008
	Strongly non-informative	0.2543	–0.0073	–0.0023
0.80	Strongly informative	0.0009	0.0000	0.0000
	Informative	0.0711	–0.0025	–0.0009
	Weakly informative	0.1892	–0.0083	–0.0034
	Weakly non-informative	0.2769	–0.0139	–0.0061
	Strongly non-informative	0.4361	–0.0233	–0.0116

## Discussion

The central assumption of LCA is the conditional independence of indicators, given latent class membership. The current literature has shown that Bayesian LCA under the assumption of approximate independence provides an accessible alternative way of detecting violations of the conditional independence assumption (Asparouhov and Muthén, 2011). Unfortunately, little is known about how the performance of Bayesian LCA would be changed by a different choice of prior, even though the prior is the key element of Bayesian analysis. The current study, therefore, explores the utility of priors in a range of prior variances in terms of Type I error and power for detecting conditional dependence, model fit (PPP), and parameter bias. In doing so, the authors believe this article contributes to the methodology and applied communities by offering modeling guidance to be considered when researchers choose Bayesian LCA as a tool for analyzing highly correlated data.

### Summary of Findings and Implications

The findings of the current simulation study show that Bayesian LCA could adequately control for the Type I error of falsely finding a true zero correlation as significantly different from zero. In fact, it is a somewhat rigorous test with Type I error smaller than 5% for all conditions examined. Power for detecting a nonzero correlation increased if a non-informative, rather than informative, prior was chosen. If the researcher secured a sample that included 300 or more, power for testing a large correlation would be satisfactory with any choice from weakly informative to strongly non-informative priors. For all priors examined, a true small correlation (i.e., 0.20) was significant in less than half of the replications. This finding implies that even when a correlation is estimated to have a positive value, the modeling may not produce enough power to establish significance for that correlation. Thus, a non-significant correlation should not be automatically discounted as being zero. Instead, the size of the estimated value should also be taken into account (Ulbricht et al., 2018). Unfortunately, another layer of complexity is that the Bayesian estimate may be biased downwardly by the presence of other nonzero correlations among the indicators, as observed in the current simulation. In many research settings, the true distribution of parameters is usually unknown and thus, researchers should be cautious about choosing extremely informative priors in either direction.

This study also found that model fit (PPP) of Bayesian LCA is susceptible to the magnitude of conditional dependence and the prior variance specified for the corresponding parameters (correlations). It is not surprising that in our simulation, approximate independence models fit well when the actual value of indicator correlations was equal to the prior mean (0). Rather, it is interesting that when the actual correlation value was different from the prior mean, model fit decreased as a more informative prior (i.e., smaller prior variance) was imposed on the correlations. A smaller variance may not let correlations escape from their zero prior mean, producing a worse PPP value. In a similar vein, model fit was acceptable when a non-informative prior was specified with a large prior variance regardless of the degree of dependence.

One should determine priors ahead of data collection in accordance with his/her substantive theory and/or previous findings from similar populations. In the context of cluster analysis, the researcher may consider either informative or non-informative priors when conditional independence has been confirmed a priori so that indicator correlations are nuisance parameters. More often, the nature of cluster analysis is rather exploratory (Lanza and Cooper, 2016) looking for or testing for correlated indicators. In such a case, the researcher may begin with non-informative priors reflecting large uncertainty on the parameter values. Otherwise, a range of priors will be equally inspired. Nevertheless, our simulation suggests that less informative priors, even strongly non-informative priors, would be a promising choice for running flexible Bayesian LCA.

### Limitations and Future Research

Although a few important findings and implications were discussed in this article, the current simulation study has two notable limitations that need to be addressed in future research. First, one must assess the validity of the findings because any variation in Bayesian application may affect the trustworthiness of the simulation results. For instance, other BSEM fit measures-e.g., deviance information criteria (Spiegelhalter et al., 2002) widely applicable information criterion (Watanabe, 2010) and leave-one-out cross-validation statistics (Gelfand, 1996) and available significance tests for conditional independence (Andrade et al., 2014) should be considered to confirm the performance of flexible Bayesian LCA.

Second, caution should be paid to generalizing the findings beyond the conditions included in the study. The simulation considered only two latent classes and a relatively small number (10) of binary indicators. The number of latent classes and the number of their indicators are not expected to considerably affect Type I error and power of the analysis and bias in parameter estimation but may have an impact on model fit (PPP). In addition, only the default priors set by M*plus* 8 were analyzed in this study. Asymmetric, rather than symmetric, binomial distribution may be a better prior for correlations among class indicators because correlations are bounded by two values (–1 and 1). Mode or mean of posterior distribution, rather than median, can serve as a better point estimate for correlations particularly when the distribution is not normal. Because the exact distribution of a posterior is typically not known, it is recommended to plot the posterior distribution and choose the measure that best represents the sample. Taken together, further simulation work is encouraged to continue to increase the utility of Bayesian LCA for various models and data environments.

## Data Availability Statement

The datasets generated for this study are available upon request.

## Author Contributions

All authors substantially contributed to the conception or design of the work and analysis and interpretation of data for the work.

## Conflict of Interest

The authors declare that the research was conducted in the absence of any commercial or financial relationships that could be construed as a potential conflict of interest.

## Appendix

**Table:**

TITLE:	Bayesian latent class analysis model of approximate independence
DATA:	FILE = example.dat; !Names data set.
VARIABLE:	NAMES = u1-u10; !Assigns names to the variables in the data set CATEGORICAL = u1-u10; !Specifies which dependent variables are treated as binary or ordered categorical variables in the model and its estimation. In this example, all dependent variables are binary. MISSING =.; !Specifies the values or symbols in the data set that are treated as missing or invalid. In this example, dot (.) is the missing value flag. CLASSES = C(2);!Assigns names to the categorical latent variables in the model and specifies the number of latent classes for each categorical latent variable. In this example, the latent variable C has two classes.
ANALYSIS:	ESTIMATOR = BAYES; !Activates the Bayesian estimator. CHAINS = 2; !Specifies using two Markov Chains for conducting the analysis. PROCESSORS = 2; !For use in multi-core systems, assigns two processors with one per chain; TYPE = MIXTURE; !Carries out a mixture analysis.
MODEL:	%OVERALL% %C#1% !In the first class, [u1$1-u10$1*1]; !Specifies starting values (in this example, 1) for the thresholds of the dependent variables u1-u10 WITH u1-u10*0 (p1-p45); !Assigns labels to the correlations among the dependent variables %C#2% [u1$1-u10$1*1]; u1-u10 WITH u1-u10*0 (p46-p90);
MODEL PRIORS:	p1-p90 ∼ IW(0, 11); !Assigns priors (in this example, inverse Wishart distributions) to the correlations among the dependent variables.

## References

[B1] Albert P. Dodd L. (2004). A cautionary note on the robustness of latent class models for estimating diagnostic error without a gold standard. *Biometrics* 60 427–435. 10.1111/j.0006-341x.2004.00187.x 15180668

[B2] Andrade P. D. Stern J. M. Pereira C. A. (2014). Bayesian test of significance for conditional independence: the multinomial model. *Entropy* 16 1376–1395. 10.3390/e16031376

[B3] Asparouhov T. Muthén B. (2010). *Bayesian Analysis Of Latent Variable Models Using Mplus.* Technical report Los Angeles, CA: Muthén & Muthén.

[B4] Asparouhov T. Muthén B. (2011). Using Bayesian priors for more flexible latent class analysis. *Proc. Joint Statist. Meet.* 2011 4979–4993.

[B5] Asparouhov T. Muthén Â (2014). Auxiliary variables in mixture modeling: three-step approaches using Mplus. *Struct. Equ. Model.* 21 329–341. 10.1080/10705511.2014.915181

[B6] Barnard J. McCulloch R. Meng X. L. (2000). Modeling covariance matrices in terms of standard deviations and correlations, with applications to shrinkage. *Statist. Sin.* 10 1281–1311.

[B7] Gelfand A. E. (1996). “Model determination using sampling-based methods,” in *Markov Chain Monte Carlo in Practice*, eds Gilks W. R. Richardson S. Spiegelhalter D. J. (London: Chapman & Hall), 145–162.

[B8] Gelman A. (2002). “Prior distribution,” in *Encyclopedia of Environmetrics*, Vol. 3 eds El-Shaarawi A. H. Piegorsch W. W. (Chichester: John Wiley and Sons), 1634–1637.

[B9] Gelman A. Meng X. L. Stern H. S. Rubin D. B. (1996). Posterior predictive assessment of model fitness via realized discrepancies. *Statist. Sin.* 6 733–807.

[B10] Gelman A. Rubin D. B. (1992). Inference from iterative simulation using multiple sequences (with discussion). *Statist. Sci.* 7 457–511.

[B11] Geweke J. Amisano G. (2011). Hierarchical Markov normal mixture models with applications to financial asset returns. *J. Appl. Econometr.* 26 1–29. 10.1002/jae.1119

[B12] Gill J. (2008). *Bayesian Methods: A Social And Behavioral Sciences Approach.* New York, NY: Chapman & Hall.

[B13] Hagenaars J. A. (1988). Latent structure models with direct effects between indicators local dependence models. *Sociol. Methods Res.* 16 379–405. 10.1177/0049124188016003002

[B14] Hansen M. Cai L. Monroe S. Li Z. (2016). Limited-information goodness-of-fit testing of diagnostic classification item response models. *Br. J. Math. Statist. Psychol.* 69 225–252. 10.1111/bmsp.12074 27404336

[B15] Im K. S. (2017). *The Hierarchical Testlet Response Time Model: Bayesian Analysis Of A Testlet Model For Item Responses And Response Times.* Doctoral dissertation, University of Kansas, Lawrence, KS.

[B16] Lanza S. T. Cooper B. R. (2016). Latent class analysis for developmental research. *Child Dev. Perspect.* 10 59–64. 10.1111/cdep.12163 31844424 PMC6914261

[B17] Lazarsfeld P. F. Henry N. W. (1968). *Latent Structure Analysis.* Boston, MA: Houghton Mifflin.

[B18] Li Y. Lord-Bessen J. Shiyko M. Loeb R. (2018). Bayesian Latent Class Analysis Tutorial. *Multivar. Behav. Res.* 53 430–451. 10.1080/00273171.2018.1428892 29424559 PMC6364555

[B19] Lubke G. Muthén B. (2007). Performance of factor mixture models as a function of model size, criterion measure effects, and class-specific parameters. *Struct. Equ. Model.* 14 26–47. 10.1080/10705510709336735

[B20] MacCallum R. C. Edwards M. C. Cai L. (2012). Hopes and cautions in implementing Bayesian structural equation modeling. *Psychol. Methods* 17 340–345. 10.1037/a0027131 22962888

[B21] Magidson J. Vermunt J. K. (2004). “Latent class models,” in *Handbook Of Quantitative Methodology For The Social Sciences*, ed. Kaplan D. (Newbury Park, CA: Sage), 175–198.

[B22] McLachlan G. J. Do K. A. Ambroise C. (2004). *Analyzing Microarray Gene Expression Data.* Hobokin, NJ: Wiley.

[B23] Muthén B. Asparouhov T. (2012). Bayesian structural equation modeling: a more flexible representation of substantive theory. *Psychol. Methods* 17 313–335. 10.1037/a0026802 22962886

[B24] Muthén B. O. (2002). Beyond SEM: general latent variable modeling. *Behaviormetrika* 29 81–117. 10.2333/bhmk.29.81 17170385

[B25] Muthén B. O. Shedden K. (1999). Finite mixture modeling with mixture outcomes using the EM algorithm. *Biometrics* 55 463–469. 10.1111/j.0006-341x.1999.00463.x 11318201

[B26] Oberski D. L. van Kollenburg G. H. Vermunt J. K. (2013). A monte carlo evaluation of three methods to detect local dependence in binary data latent class models. *Adv. Data Analy. Classif.* 7 267–279. 10.1007/s11634-013-0146-2

[B27] O’sullivan A. M. (2013). *Bayesian Latent Variable Models With Applications.* Doctoral dissertation, Imperial College London, London.

[B28] Qu Y. Tan M. Kutner M. (1996). Random effects models in latent class analysis for evaluating accuracy of diagnostic tests. *Biometrics* 52 797–810. 8805757

[B29] Rupp A. Templin J. Henson R. (2010). *Diagnostic Measurement: Theory, Methods, And Applications.* New York, NY: Guilford Press.

[B30] Scheines R. Hoijtink H. Boomsma A. (1999). Bayesian estimation and testing of structural equation models. *Psychometrika* 64 37–52. 10.1007/bf02294318

[B31] Schlattmann P. (2010). *Medical Applications Of Finite Mixture Models.* Berlin, Germany: Springer.

[B32] Silva R. Ghahramani Z. (2009). The hidden life of latent variables: bayesian learning with mixed graph models. *J. Mach. Learn. Res.* 10 1187–1238.

[B33] Spiegelhalter D. J. Best N. G. Carlin B. Van der Linde A. (2002). Bayesian measures of model complexity and fit. *J. R. Statist. Soc. Ser. B* 64 583–639. 10.1111/1467-9868.00353

[B34] Torrance-Rynard V. Walter S. (1998). Effects of dependent errors in the assessment of diagnostic test performance. *Statist. Med.* 16 2157–2175. 10.1002/(sici)1097-0258(19971015)16:19<2157::aid-sim653>3.0.co;2-x 9330426

[B35] Uebersax J. S. (1999). Probit latent class analysis with dichotomous or ordered category measures: conditional independence/dependence models. *Appl. Psychol. Measur.* 23 283–297. 10.1177/01466219922031400

[B36] Ulbricht C. M. Chrysanthopoulou S. A. Levin L. Lapane K. L. (2018). The use of latent class analysis for identifying subtypes of depression: a systematic review. *Psychiatr. Res.* 266 228–246. 10.1016/j.psychres.2018.03.003 29605104 PMC6345275

[B37] Vacek P. (1985). The effect of conditional dependence on the evaluation of diagnostic tests. *Biometrics* 41 959–968. 3830260

[B38] Vermunt J. K. (2010). Latent class modeling with covariates: two improved three-step approaches. *Polit. Analy.* 18 450–469. 10.1093/pan/mpq025

[B39] Wang W.-C. Wilson M. (2005). The Rasch testlet model. *Appl. Psychol. Measur.* 29 126–149. 10.1177/0146621604271053

[B40] Watanabe S. (2010). Asymptotic equivalence of Bayes cross validation and widely applicable information criterion in singular learning theory. *J. Mach. Learn. Res.* 11 3571–3594.

[B41] Yang M.-H. Ahuja N. (2001). *Face Detection And Gesture Recognition For Human-Computer Interaction.* Boston, MA: Springer.

[B42] Zhan P. Liao M. Bian Y. (2018). Joint testlet cognitive diagnosis modeling for paired local item dependence in response times and response accuracy. *Front. Psychol.* 9:607. 10.3389/fpsyg.2018.00607 29922192 PMC5996944

